# A Comparative Study between Laparoscopic Sleeve Gastrectomy and One Anastomosis Gastric Bypass on Serum Zinc Levels

**DOI:** 10.1007/s11695-025-08422-2

**Published:** 2025-12-17

**Authors:** Ahmed Mohammed Salah Eldeen Othman Elansary, Mohamed Hassan Ali Fahmy, Shady Othman Rmadan Elsayed, Mohamed Yacoub, Abdelrahman M. Mohamed

**Affiliations:** 1 General Surgery Department, Faculty of Medicine, Cairo University, Cairo, Egypt; 2 General Surgery Department, Nasr City Health Insurance Hospital, Cairo, Egypt

## Abstract

**Introduction:**

Bariatric surgery effectively induces weight loss, yet its impact on micronutrient homeostasis, particularly zinc, remains unclear. Given zinc’s essential role in wound healing, immunity, and hair growth, understanding postoperative changes is clinically relevant. This study evaluated the effect of sleeve gastrectomy and one-anastomosis gastric bypass on serum zinc levels.

**Methods:**

The prospective cohort study recruited 50 patients with severe obesity who underwent bariatric surgery. Patients were divided to two groups and monitored for one year. Laparoscopic sleeve gastrectomy group included 25 participants; one anastomosis gastric bypass surgery group included 25 participants. Primary outcome was the assessment of serum zinc levels at 3, 6, and 12 months postoperatively. Secondary outcomes included the incidence of symptoms associated with reduced zinc levels, such as hair loss, diarrhea, and delayed wound healing.

**Results:**

Patients who underwent one-anastomosis single bypass surgery had a higher incidence of low serum zinc levels at 6 months and at 12 months postoperatively compared to those who underwent sleeve gastrectomy (60.5 ± 20.9 vs. 79.8 ± 19.1 µg/dL, *p* = 0.008, and 49.7 ± 19.1 vs. 79.2 ± 22.8 µg/dL, *p* < 0.001). Symptoms related to hypozincemia, including hair loss, diarrhea and delayed wound healing, were significantly increased after one-anastomosis single bypass surgery (*p* ˂ 0.05).

**Conclusion:**

One-anastomosis single bypass is associated with a higher postoperative incidence of hypozincemia, as well as a worsening of symptoms compared to sleeve gastrectomy.

## Key Points

• Bariatric surgery alters micronutrient balance, including zinc levels.

• Zinc levels dropped more after bypass than sleeve gastrectomy.

• Hypozincemia symptoms were more common in the bypass group.

• Routine zinc monitoring is essential after bariatric surgery.

## Introduction

Obesity is a major public health issue associated with increased morbidity, mortality, and reduced quality of life. It contributes to an estimated 2.8 million deaths annually worldwide due to its link with multiple comorbid conditions [1]. Beyond excessive fat accumulation, individuals with obesity frequently exhibit vitamin and micronutrient deficiencies, partly as a result of chronic inflammation, poor diet quality, and altered metabolism [2]. These deficiencies may worsen after bariatric surgery, which—despite being the most effective and durable treatment for severe obesity—can further disrupt nutrient absorption and utilization [3, 4].

 Micronutrient homeostasis, particularly involving trace elements such as zinc, plays a vital role in maintaining metabolic health after bariatric surgery. Zinc is essential for numerous biological processes, including cellular metabolism, immune defense, wound healing, and epithelial integrity [2, 5]. Its absorption occurs mainly in the duodenum and proximal jejunum, making it susceptible to postoperative alterations depending on the surgical technique. Consequently, zinc deficiency has emerged as one of the most relevant and underrecognized complications following bariatric surgery [6].

Among various procedures, laparoscopic sleeve gastrectomy (SG) and one-anastomosis gastric bypass (OAGB) are currently among the most widely performed operations worldwide [4, 7]. SG is technically straightforward and effective in inducing weight loss, but concerns persist regarding long-term outcomes and postoperative reflux [8]. Conversely, OAGB is associated with excellent weight loss and comorbidity resolution; however, its malabsorptive component may predispose patients to a greater risk of micronutrient deficiencies, including zinc depletion [7, 8].

Recognizing this, professional guidelines such as those from the American Society for Metabolic and Bariatric Surgery (ASMBS) and the British Obesity and Metabolic Surgery Society (BOMSS) recommend routine postoperative monitoring and supplementation of zinc. Nevertheless, the optimal supplementation regimen, frequency of monitoring, and comparative impact of different bariatric procedures on zinc levels remain poorly defined [9].

Therefore, the present study aimed to evaluate and compare the effects of sleeve gastrectomy and one-anastomosis gastric bypass on serum zinc levels, thereby contributing to a better understanding of postoperative micronutrient management in bariatric surgery patients.

## Materials and Methods

### Ethical Considerations

All procedures performed in studies involving human participants were in accordance with the ethical standards of the institutional and/or national research committee and with the 1964 Helsinki declaration and its later amendments or comparable ethical standards. Informed consent was obtained from all individual participants included in the study. The privacy of every participant’s information was assured.

### Study Design, Setting and Location

This prospective cohort study was conducted at the General Surgery Department, the University Hospitals.

### Eligibility Criteria

The study population included patients of both genders who were aged 18 years or older, had a BMI of 35 kg/m^2^ or higher without associated medical problems, or 30 kg/m^2^ with associated medical problems. These patients underwent laparoscopic sleeve gastrectomy or one-anastomosis gastric bypass surgery under general anesthesia. Patients were **not randomly assigned** to either procedure. The choice between sleeve gastrectomy (SG) and one-anastomosis gastric bypass (OAGB) was made according to preoperative multidisciplinary evaluation, which considered factors such as baseline body mass index (BMI), presence of gastroesophageal reflux disease, type 2 diabetes, and patient preference after detailed counseling about each procedure’s risks and benefits. All operations were performed by the same experienced surgical team following standardized techniques. Patients under 18 years of age, pregnant patients, and those deemed unsuitable for general anesthesia were excluded from the study. Also, patients afflicted with severe associated medical problems, including human immunodeficiency virus, cancer, hepatitis C virus, cerebrovascular diseases, or exhibiting severe mental or cognitive disorders were excluded.

Preoperative evaluation included assessment of other key micronutrients such as iron, vitamin B12, folate, vitamin D, and copper. Patients found to have abnormal levels of any of these micronutrients were excluded from the study to minimize confounding effects. However, these additional micronutrients were not followed postoperatively, as the primary objective of the study was to assess changes in serum zinc level.

### Preoperative Assessment

A comprehensive evaluation was conducted to assess the patients’ general health, associated medical problems, risk factors, psychological condition, capacity to adhere to a postoperative regimen, and preoperative symptoms of serum zinc deficiency, such as hair loss, diarrhea, glossitis, nail dystrophy, delayed wound healing, skin lesions, and neuropsychological disturbances. Laboratory tests were performed, including preoperative serum zinc level. We excluded the cases with micronutrient deficiencies including zinc from this study.

### Operative Details

Patients were divided to two groups. The sleeve gastrectomy group (SG) included 25 patients with all cases 36 f bougie was used, and the one-anastomosis gastric bypass group (OAGB) included 25 patients with biliopancreatic length ranged from 180 to 200 cm. All patients received general anesthesia.

### Postoperative Care

Subsequent follow-ups were conducted at three months, six months, and one year after the initial procedure. All patients were advised to take daily oral supplements, including low-dose zinc supply (5 mg) for a minimum of 1 year in the gastric sleeve and for life in OAGB. At 3, 6, and 12 months postoperatively, all patients were evaluated with serum zinc levels and symptoms of serum zinc deficiency. Postoperative clinical manifestations, including hair loss, diarrhea, poor wound healing, and neuropsychological symptoms, were recorded during scheduled follow-up visits using a structured symptom checklist. Data were primarily self-reported and verified by clinical evaluation when applicable.”

### Study Outcomes

The primary outcome was postoperative serum zinc levels. Secondary outcomes included the incidence of postoperative zinc deficiency symptoms.

### Sample Size

According to Krzizek et al. [9], serum zinc level was between 0.54 and 1.03 mg/L before BS. A rise of serum zinc deficiency of about 1%–1.7% was noted after the procedure. The sample size consisted of 50 BS patients with a 100% follow-up rate, and it was calculated to evaluate the effect of bariatric surgeries on serum zinc levels. The Sample Size Calculator was used to establish the alpha error at 0.05, the power at 0.80, and the confidence interval at 95%.

### Statistical Analysis

The Statistics Package for Social Sciences (SPSS) software, version 25.0, was used. Category data was presented as percentages and numerical values. The Shapiro-Wilk test was used to assess the normality of continuous data. Quantitative data were reported using the range, mean, and standard deviation (SD). The chi-square test and independent Student’s t-test were used to evaluate the relationship between two sets of qualitative variables and quantitative data with normal distributions, respectively. A 5% alpha criterion was set for every statistical test.

## Results

A total of 50 patients were divided into two groups: 25 underwent laparoscopic SG and 25 underwent OAGB surgeries. There were no significant differences in the demographic characteristics of the groups with regard to age, gender, weight, height, or BMI (Table 1).

**Table 1:** Patients’ baseline characteristics (*n* = 50)

		Sleeve Gastrectomy (*n* = 25)	One anastomosis single bypass (*n* = 25)	Stat. test	*P*-value
Age, years	Mean ± SD	33.6 ± 11	38.8 ± 9.6	1.59	0.21
	Min – Max	18–68	21–57		
Gender, *n* (%)	Male	5 (20.0%)	9 (36.0%)	-1.78	0.081
	Female	20 (80.0%)	16 (64.0%)		
Weight	Mean ± SD	124.3 ± 20	128.9 ± 27.9	-0.673	0.504
	Min – Max	94–180	93–188		
Height	Mean ± SD	161.8 ± 6.1	162.8 ± 17.1	-0.27	0.788
	Min – Max	154–176	104–185		
BMI	Mean ± SD	47.5 ± 6.6	47.7 ± 13.1	-0.056	0.956
	Min – Max	38.1–60	34.5–83.6		

*BMI* body mass index, *n* number, *SD* standard deviation, *Min* minimum, *Max* maximum

All patients had normal preoperative levels of iron, vitamin B12, folate, vitamin D, and copper. Patients with abnormal results were excluded from the study. Postoperative follow-up focused exclusively on serum zinc levels, and other micronutrient measurements were not routinely performed.

Prior to undergoing the surgical procedure and at the 3-month postoperative stage, the serum zinc levels of the two groups did not demonstrate any significant differences. At 6 and 12 months postoperatively, serum zinc levels were significantly reduced in the OAGB group compared to the sleeve gastrectomy group (*p* = 0.001 and **<** 0.001, Table 2).

**Table 2:** Preoperative and postoperative zinc levels (*n* = 50)

Zinc level (µg/dL)		Sleeve gastrectomy (*n* = 25)	One anastomosis single bypass (*n* = 25)	T	*P*-value
Before surgery	Mean ± SD	83.4 ± 18.2	86.5 ± 20.5	-0.569	0.57
	Min – Max	59–120	60–150		
After 3 months	Mean ± SD	81.4 ± 17.8	75.6 ± 24.9	0.946	0.349
	Min – Max	55–120	40–155		
After 6 months	Mean ± SD	79.8 ± 19.1	60.5 ± 20.9	3.41	0.001 *
	Min – Max	56–117	31–105		
After 12 months	Mean ± SD	79.2 ± 22.8	49.7 ± 19.1	4.95	< 0.001 *
	Min – Max	12–116	20–103		

*n* number, *SD* standard deviation, *Min* minimum, *Max* maximum, *T* independent sample T test; * significant at *p* < 0.05

Compared to the sleeve gastrectomy group, patients in the OAGB group exhibited a significant increase in postoperative hair loss, neuropsychological symptoms, and diarrhea, accompanied by a significant reduction in wound healing (*p* < 0.001, Table 3).

**Table 3:** Postoperative symptoms after one year (*n* = 50)

		Sleeve gastrectomy (*n* = 25)		One anastomosis single bypass (*n* = 25)		X^2^	*P*-value
Hair loss	No	25	100.0%	12	48.0%	17.6	< 0.001*
	Yes	0	0.0%	13	52.0%		
Neuro-psychological symptoms	No	25	100.0%	15	60.0%	12.5	< 0.001 *
	Yes	0	0.0%	10	40.0%		
Diarrhea	No	25	100.0%	13	52.0%	15.8	< 0.001 *
	Yes	0	0.0%	12	48.0%		
Decreased Wound healing	No	25	100.0%	14	56.0%	14.1	< 0.001 *
	Yes	0	0.0%	11	44.0%		

*n* number, *X*^2^ chi-square test; * significant at *p* < 0.05

## Discussion

Despite the fact that BS is a safe and successful solution for individuals who have severe obesity, there is conflicting data regarding its impact on serum zinc levels. This study aimed to assess the effect of bariatric surgeries on serum zinc levels in patients undergoing SG or OAGB.

The primary findings of this study demonstrate that patients in the OAGB group exhibited a substantial decrease in zinc levels at 6 and 12 months following surgery, accompanied by a notable exacerbation of the clinical manifestations associated with hypozincemia.

Correspondingly, Moize et al. [10] and Skroubis et al. [11] found that surgeries, such as SG, are less likely to result in micronutrient deficiencies than techniques, such as OAGB. Robert et al. [12] noted an increased risk of nutritional deficits after OAGB with a 200 cm biliopancreatic limb at two-year follow-up. Furthermore, after a five-year follow-up, Plamper et al. [5] found that OAGB had a higher prevalence of nutritional deficits than SG. Furthermore, in a systematic review and meta-analysis, Jiao et al. [13] found that individuals with obesity have zinc insufficiency before surgery and experience worsened symptoms after surgery, even after multivitamins and trace elements were routinely supplemented.

Mahawar et al. [14] reported that more than half of the patients had zinc insufficiency prior to receiving BS. The precise mechanisms causing the drop in serum zinc levels following BS are still unclear. However, avoiding primary absorption channels, early gut mucosal injury, and inadequate nutrition are potential causes. Bariatric surgery patients may have a saturated zinc absorption capacity due to their poor absorption ability.

A zinc deficiency in the bloodstream increases the risk of certain metabolic disorders. Fukunaka and Fujitani [15] reported that zinc deficiency may increase the prevalence of diabetes and is associated with the pathophysiology of type 2 diabetes. These results highlight the importance of vigilant monitoring and supplementation after surgery and underscore the significance of zinc in human physiological processes.

Three months after RYGB surgery, Rosa’s research found that patients’ serum zinc concentrations dropped dramatically after receiving a single 15 mg oral zinc supplement dosage. Serum zinc’s area under the curve decreased by 89% [16]. Moreover, Ruz et al. [17] reported that zinc absorption rate dropped from 32.3% before surgery to 13.6% six months afterward, then rose to 21% eighteen months later. Clearly, RYGB surgery results in decreased zinc absorption, as well as decreased dietary zinc intake. Ruz and colleagues contend that a daily zinc dose of 9.5 mg was insufficient to prevent zinc deficiency following RYGB surgery. Higher dosages of 40–60 mg/day were recommended. However, the acceptable maximum consumption limit of zinc is 40 mg/day.

According to Sallé et al. [18], 42.5% of patients still had a zinc deficiency one year after surgery, even after taking a daily zinc supplement of 15 mg. These findings raise the possibility that postoperative zinc supplementation requirements for patients were overestimated.

According to the BOMSS guidelines, it is recommended that all patients who undergo BS should take zinc supplements [8]. For patients undergoing LSG and RYGB, the recommended initial dose is 15 mg/day. For patients with additional needs, such as those undergoing biliopancreatic diversion with duodenal switch (BPD/DS), the recommended initial dose is 30 mg/day. While supplementing with zinc, it is important to pay attention to the supplementation of copper [19, 20]. However, excessive zinc supplementation can lead to zinc toxicity and interfere with the absorption of other essential elements like iron and copper. Therefore, the suggestion to supplement such high doses of zinc may not be appropriate and needs careful consideration.

In the present study, 35% of patients who underwent OAGB reported memory loss, 55% experienced diarrhea, 35% exhibited impaired taste and smell, 45% demonstrated delayed wound healing, and 50% reported hair loss. These results are consistent with Mahawar et al. [14] that showed different BS procedures may cause varied hypozincemia-related symptoms. Gunstad et al. [21] reported that one anastomosis bypass operation showed higher cognitive variability compared to more limited surgical techniques.

However, Plamper et al. [5] found no difference in hypozincemia symptoms between the OAGB and SG groups. Also, Mohamed Deabes et al. [22] found that there was no discernible difference in complications between the laparoscopic single anastomosis sleeve ileum bypass (SASI bypass) versus laparoscopic mini-gastric bypass. Moreover, Vilallonga et al. [23] did not discover any statistically significant differences between patients with obesity with BMI >50 or BMI˂50 concerning immediate or long-term complications after laparoscopic sleeve gastrectomy.

Although our results demonstrated a significant postoperative decline in serum zinc and a higher frequency of hair loss in the OAGB group, this relationship should be interpreted with caution. Hair loss following bariatric surgery is multifactorial and may also be influenced by rapid weight reduction, caloric restriction, protein deficiency, hormonal changes, and deficiencies in iron, biotin, vitamin B12, folate, and vitamin D. Therefore, the observed association between zinc deficiency and hair loss in our cohort does not establish causation but highlights the need for comprehensive micronutrient monitoring after bariatric surgery.

### Limitations

It is imperative to consider the limitations of the study when interpreting the results. The study’s relatively small sample size of 50 individuals and single-center design may have limited the extent to which the results can be generalizable. A year following the surgical procedure, the study does not provide a comprehensive evaluation of serum zinc levels over an extended time period. To further expand our understanding in this domain, it is imperative to conduct additional extensive multicenter trials with prolonged follow-up periods.

Although patients with preoperative deficiencies of other micronutrients were excluded, postoperative assessment focused solely on zinc. As several micronutrient deficiencies can produce similar clinical symptoms, these manifestations cannot be exclusively attributed to zinc deficiency.

Postoperative zinc deficiency is likely multifactorial, potentially influenced by reduced dietary intake, medication use, and underlying medical conditions that affect absorption or metabolism. While our study focused on procedure-related changes, these contributing factors should be considered when interpreting the findings.

## Conclusions

Compared to sleeve gastrectomy, patients who underwent OAGB surgery have been associated with a significantly higher incidence of postoperative hypozincemia and a worsening of postoperative symptoms. These results underscore the importance of customized surgical strategies, comprehensive nutritional evaluation prior to surgery, and strict postoperative surveillance. The necessity of customized nutritional management plans and patient education regarding procedure-specific risks and long-term metabolic consequences is indisputable.

## Data Availability

The data supporting the findings of this study are contained within the article.
